# MRI-TRUS fusion targeted biopsy is highligheted in International Brazilian Journal of Urology

**DOI:** 10.1590/S1677-5538.IBJU.2023.03.01

**Published:** 2023-05-26

**Authors:** Luciano A. Favorito

**Affiliations:** 1 Unidade de Pesquisa Urogenital Universidade do Estado do Rio de Janeiro Rio de Janeiro RJ Brasil Unidade de Pesquisa Urogenital - Universidade do Estado do Rio de Janeiro - Uerj, Rio de Janeiro, RJ, Brasil,; 2 Serviço de Urologia Hospital Federal da Lagoa Rio de Janeiro RJ Brasil Serviço de Urologia, Hospital Federal da Lagoa, Rio de Janeiro, RJ, Brasil

The May-June number of Int Braz J Urol is the 22^nd^ under my supervision. In this number the Int Braz J Urol presents original contributions with a lot of interesting papers in different fields: Robotic Surgery, Prostate Cancer, Endometriosis, Translational Research, Male Health and Renal stones, Kidney Cancer, Bladder Cancer and UPJ obstruction. The papers came from many different countries such as Brazil, Italy, China, Saudi Arab and USA, and as usual the editor´s comment highlights some of them. The editor in chief would like to highlight the following works:

Dr. Wang and collegues from China, presented in page 281 (1) a nice systematic review about the impact of body mass index (BMI) on size and composition of urinary stones and concluded that the current evidence suggests a positive association between BMI and uric acid and calcium oxalate stones. It would be of great guiding significance to con- sider losing weight when treating and preventing urinary stones.

Dr. Ribeiro-Julio and collegues from Brazil, presented in page 299 (2) a important review about the anatomy of the lower hypogastric plexus applied to endometriosis and concluded that the Accurate knowledge of the innervation of the female pelvis is of fundamental importance for prevention of possible injuries and voiding dysfunctions as well as the evacuation mechanism in the postoperative period. Imaging exams such as nuclear magnetic resonance are interesting tools for more accurate visualization of the distribution of the hypogastric plexus in the female pelvis.

Dr. da Silva and collegues from Brazil, performed in page 320 (3) a interesting translational research about the effects of dutasteride and tamsulosin on penile morphology in a rodent model and concluded that both treatments with dutasterid and tamsulosin promoted penile morphometric modifications in a rodent model. The combination therapy resulted in more notable modifications. The results of this study may help to explain the erectile dysfunction observed in some men using these drugs.

Dr. Moratti Gilberto and collegues from Brazil performed in page 334 (4) an interesting study about the complication rates of transrectal and transperineal prostate fusion biopsies in a high-volume interventional center and concluded that the learning curve for performing the transperineal biopsy, with a lower rate of complications for the experienced team, after 142 cases after 6 months of practice. The lower complication rate of transperineal prostate biopsy and the absence of infectious prostatitis imply a safer procedure when compared to transrectal prostate biopsy.

Dr. Raed and collegues from Saudi Arab permormed in page 372 (5) a nice study about the influence of 3D renal reconstruction on surgical planning for complex renal tumors and concluded that customized interactive virtual 3D models seem to provide superior visualization of the anatomical details and pathologic morphology of complex renal tumors over traditional visualization methods. Therefore, the surgeon can appropriately plan and modify the proposed surgical strategy, especially when minimally invasive partial nephrectomy is considered.

Dr. Sobhani and collegues from USA permormed in page 351 (6) a nice study about the perioperative mortality for radical cystectomy (RC) and conclude that the 90-day mortality for RC is approaching five percent, with infectious, pulmonary, and cardiac complications as the leading mortality causes. Older age, higher comorbidity, blood transfusion, and pathological lymph node involvement are independently associated with 90-day mortality.

Dr. Lv and collegues from China performed in page 359 (7) the cover paper of this edition. In this paper the authors studied if is it necessary for all patients with suspicious lesions undergo systematic biopsy in the era of MRI-TRUS fusion targeted biopsy and concluded that the mean PSA density (PSAD) combined with PI-RADS showed utility in guiding optimization of the prostate biopsy mode. Higher PSAD and PI-RADS values were associated with greater confidence in implementing mono-Targeted biopsy and safely omitting systematic biopsy, thus effectively balancing the benefits and risks.

The Editor-in-chief expects everyone to enjoy reading.
